# The Emergence of Environmental Homeostasis in Complex Ecosystems

**DOI:** 10.1371/journal.pcbi.1003050

**Published:** 2013-05-16

**Authors:** James G. Dyke, Iain S. Weaver

**Affiliations:** School of Electronics and Computer Science, University of Southampton, Southampton, United Kingdom; Princeton University, United States of America

## Abstract

The Earth, with its core-driven magnetic field, convective mantle, mobile lid tectonics, oceans of liquid water, dynamic climate and abundant life is arguably the most complex system in the known universe. This system has exhibited stability in the sense of, bar a number of notable exceptions, surface temperature remaining within the bounds required for liquid water and so a significant biosphere. Explanations for this range from anthropic principles in which the Earth was essentially lucky, to homeostatic Gaia in which the abiotic and biotic components of the Earth system self-organise into homeostatic states that are robust to a wide range of external perturbations. Here we present results from a conceptual model that demonstrates the emergence of homeostasis as a consequence of the feedback loop operating between life and its environment. Formulating the model in terms of Gaussian processes allows the development of novel computational methods in order to provide solutions. We find that the stability of this system will typically increase then remain constant with an increase in biological diversity and that the number of attractors within the phase space exponentially increases with the number of environmental variables while the probability of the system being in an attractor that lies within prescribed boundaries decreases approximately linearly. We argue that the cybernetic concept of rein control provides insights into how this model system, and potentially any system that is comprised of biological to environmental feedback loops, self-organises into homeostatic states.

## Author Summary

Life on Earth is perhaps greater than three and a half billion years old and it would appear that once it started it never stopped. During this period a number of dramatic shocks and drivers have affected the Earth. These include the impacts of massive asteroids, runaway climate change and increases in brightness of the Sun. Has life on Earth simply been lucky in withstanding such perturbations? Are there any self-regulating or homeostatic processes operating in the Earth system that would reduce the severity of such perturbations? If such planetary processes exist, to what extent are they the result of the actions of life? In this study, we show how the regulation of environmental conditions can emerge as a consequence of life's effects. If life is both affected by and affects it environment, then this coupled system can self-organise into a robust control system that was first described during the early cybernetics movement around the middle of the twentieth century. Our findings are in principle applicable to a wide range of real world systems - from microbial mats to aquatic ecosystems up to and including the entire biosphere.

## Introduction

How stable is the Earth system? If we define stability in terms of the persistence of a set of environmental conditions that are required for a widespread biosphere, then the Earth has demonstrated stability over geological time. Life emerged on Earth possibly as long as 3.7 billion years ago [1]. Since then, despite a number of planetary scale calamities such as snowball events [2], life in some form or other has continued to grow, reproduce and evolve. The original Gaia hypothesis [3] argued that this stability, rather than being the product of chance and (from our perspective) good fortune, is a demonstration of certain homeostatic properties that the Earth possesses analogous to physiological homeostasis in biological systems. This suggests there are certain differentiated processes or mechanisms operating within the Earth system that are able to oppose perturbations in such ways as to reduce their impacts. There are clear adaptive advantages to certain forms of organismic homeostasis. However, natural selection is not a mechanism that can be applied to planets. This led to the initial criticism that any form of planetary homeostasis must invoke a form of altruism on the part of the organisms involved and would therefore be subject to collapse via the inevitable emergence of ‘cheat’ organisms that reap the benefits of environmental homeostasis but without doing their fair share of the work required to maintain it [4], [5]. Despite such criticism, research into the plausibility of Gaian homeostasis has continued in the context of: natural selection [6]; ecology and evolution [7]; biogeochemical regulation [8], [9]; and complex adaptive systems [10].

Notwithstanding this work the hypothesis is still far from being confirmed. This is due in part to a certain amount of opaqueness as to exactly how such planetary homeostasis operates [11]. For example, if planetary homeostasis is the result of biological feedback on the abiotic environment, then what reasons are there to conclude that stabilising, negative feedback loops should predominate over destabilising, positive feedback loops? Some answers to this question have been proposed via the development of conceptual biosphere models such as Daisyworld [12]. The original Daisyworld was a simple model in which two different life forms (black and white ‘daisy flowers’) with opposing effects on environmental conditions grew on an Earth-like planet orbiting a Sun-like star. The feedbacks operating between the daisies and environment led to a planetary system that was homeostatic with respect to radiative forcing: as the brightness of the star increased over geological time (much like the Sun) temperatures on the surface of the planet were held within the range of temperatures required for the life forms to grow.

There have been a number of developments and extensions of Daisyworld that were in part motivated by criticisms of the original model. In addressing them it has been shown that homeostasis in conceptual planetary biosphere models can emerge with: increased biodiversity [13]; mutation and adaptation [14], [15]; higher trophic levels such as herbivory [16]; spatially explicit effects [17]. See [18] for a review of these and other studies. With regards to mechanisms that explain the homeostatic behaviour of Daisyworld, one line of argument has proposed that it is best understood as an example of a *rein control* system [19]–[21] that was mathematically developed in the context of regulation of blood glucose [22], [23]. However, while this may be a sufficient explanation for how a number of simple models of planetary homeostasis operate, there is a very large gap between them and a sufficient account for how the real and very complex Earth system may be homeostatic. Rein control systems can feature multiple variables or components that are subject to regulation, however the myriad of different feedback and inputs from diverse biological populations would seem to limit the utility of such approaches. What reasons are there to think that the homeostatic mechanisms present in such simple systems will ‘scale up’ to significantly more complex systems [24]? The danger is that while simple models may provide insights into how homeostasis may function, they risk omitting the very things from which a form of planetary homeostasis may emerge.

### Assumptions

**Figure 1:**
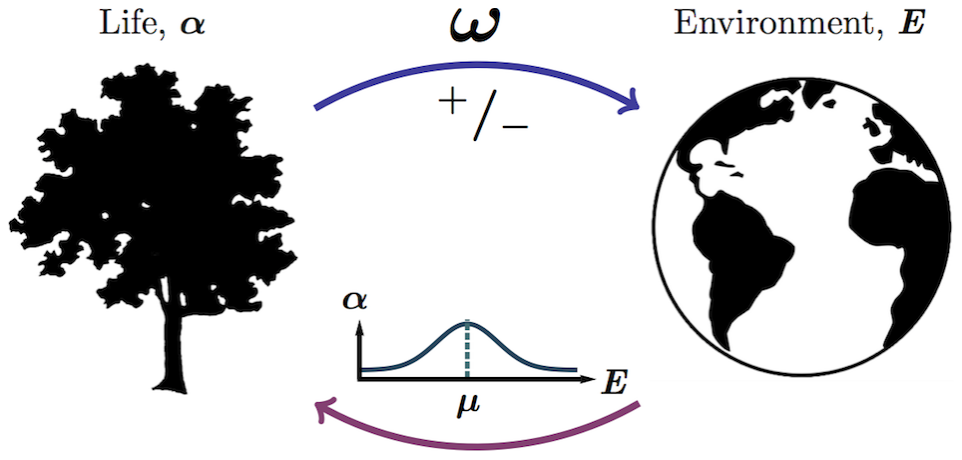
The abundance of a life, 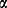 is some function of environmental conditions, *E*, parameterised by 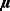. Environmental conditions are affected by the actions of life ω. While concepts such as facilitation, ecosystems engineering and niche construction include assumptions that life can alter its environmental conditions in important ways, the notion that this system self-organises into a negative feedback loop is controversial.

In considering these issues we will proceed from the most general set of assumptions and produce a model that will feature diverse biological populations that interact with an abiotic environment. In doing so we will be guided by the motivation to produce analysis and results that will in principle be of relevance to a wide range of real-world phenomena. The model is grounded on two main assumptions. First, the abundance and distribution of species is in some sense determined by environmental conditions. Second, that these same environmental conditions are in some sense determined by the abundance and distribution of species. This is represented with Figure 1.

Within theoretical ecology, the impacts of environmental conditions on life is captured by the *fundamental niche* concept [25]. The fundamental niche is the set of environmental conditions within which a particular species can grow and reproduce. This can be represented as a volume with each axis being a particular abiotic factor. This is the foundation assumption for species distribution modelling which correlates environmental conditions to the distribution and abundance of species. As environmental conditions change, either spatially or temporally, then all other things being equal, a commensurate change in the distribution of species should be observed. In the original Daisyworld, the niche axis of temperature determined the growth rate and therefore the steady state populations of the daisies. Daisies grew fastest at a particular temperature with growth rates declining as temperature deviated away from this optimal value towards the edges of the niche. A particular species will be able to exist within a particular fundamental niche, but this is not a sufficient condition for observing it in particular habitats. There will be a range of other limiting factors that means that although a particular fundamental niche may support a wide range of species, only comparatively few may be observed. This is the *realised niche* of a species [26]. Amongst the possible limiting factors is competition between different species. While species 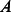 may be able to occupy a particular fundamental niche, species 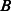 is better adapted and effectively outcompetes 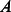 so that only 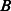 is observed within this niche. The realised niche of 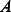 may be significantly smaller than its fundamental niche.

Being alive necessitates affecting environmental conditions if only because being alive necessitates running a metabolism and consequently the export of high entropy waste into the environment [27]. However these and other effects can lead to significant changes in environmental conditions that vary in spatial and temporal scales such as: millimetres and days in nitrogen fixing bacteria [28]; metres and decades with earthworms, plant roots and tunnelling mammals that affect the composition of soil [29], [30]; global and geological scales with changes in composition of the Earth's atmosphere as a result of oxygenic photosynthesis [31] and possible biological impacts on tectonic and Earth interior processes [32]. Within ecology and evolutionary theory, the impacts of life on its environment can be captured within the ecosystems engineering [33], and niche construction [34] concepts. Recently these and other biotic processes that can affect the distribution and abundance of species have been collected under the umbrella notion of *biotic modulator* [35].

### Structure of paper and main findings

In the section “Model” we formulate a model that features the two main assumptions. In the “Results” section we present results that show the model will, under a range of conditions, self-organise into homeostatic states that are robust to external perturbations. By developing novel computational methods based on Gaussian processes we are able to show that increasing model complexity in terms of increasing biological diversity does not decrease the homeostatic properties of the model. A more diverse system is not less stable. We will show that increasing the complexity of the model by increasing the number of environmental variables leads to an exponential increase in the number of stable points while leading to a decrease in the probability that a randomly initialised system will remain within prescribed bounds as it relaxes to a stable point. In the “Discussion” we discuss our results in the light of the relationship between complexity and stability in ecosystems as well as highlight a number of limitations of our approach and possible future work.

## Model

Let 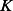 denote the number of *biotic components* in the system. A biotic component may potentially represent an individual organism, species, ecotype or population. We are intentionally vague as we do not wish to make any other assumptions about the particular forms of biotic component. Consequently, if two different species respond in the same way to an environmental variable and affect that variable in the same way, then they would be represented as a single biotic component in the model. We do not include any interactions be they trophic or competition for resources and/or space between biotic components. In that respect the fundamental niche entirely determines whether or not a particular biotic component will be present in a particular habitat. This may appear to be a crucial assumption with respect to comparisons to previous Daisyworld studies as these typically included inter-species competition between the black and white daisies in the form of a finite amount of space within which daisies would seed and grow. During periods of homeostasis, the total population of black and white daisies remained constant with the proportion of black to white daisies changing. White daisies could only increase their coverage if there was an equal amount of decrease in the coverage of the black daisies, and vice versa. We relax this assumption for the following reason: we do not wish to prescribe which limiting factors are important as competition, its strength and direction, is but one possible limiting factor; we intend our model to be in principle applicable up to the biospheric level and consequently while two or more species may occupy similar or overlapping fundamental niches, there may be no competition or other interactions between them as they may reside on different continents; the assumption of strong competition between daisies in the original Daisyworld and some of its extensions has been shown to actually *increase* the effectiveness of the homeostatic mechanism [18], [21] and so relaxing this assumption in our model makes our results and analysis more general.

Our modelled biotic component has two types of traits. The first, along with environmental conditions, determines the *abundance* variable, denoted with 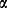, that may be understood as individual numbers or biomass or primary productivity or metabolic activity of that particular biotic component. Second, an *effect* parameter, denoted with ***ω***, that determines the biotic components impacts on aspects of its environment. The environmental conditions are represented with 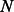 *environmental variables*, denoted with vector ***E***. We assume that the abundance of the *j*th biotic element will linearly change over time towards the steady state 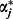:
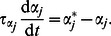
(1)As we will discuss later our results are applicable for non-linear changes towards steady state or implementations of the model that use replicator equations that feature fitness functions and fixed death rates in ways similar to the original Daisyworld model. All that matters for our analysis are the respective timescales of the biotic components and their environmental conditions. 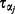 parameterises the timescale of changes in 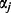. This is determined by the set of environmental conditions ***E***

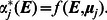
(2)The abundance of the *j*th biotic component is maximised at a point in the space of environmental variables, 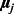. Consequently, as ***E*** departs from 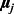, the abundance of the *j*th biotic component decreases. 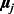 can then be understood as determining the *j*th biotic element's fundamental niche. A simple choice of niche function for 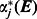 is a Gaussian centred at 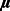 with characteristic width 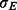. We will show later that with respect to the homeostatic properties of the model, the particular form of niche function is, subject to certain limitations, not important. For example skewed or multimodal functions produce model behaviour that is essentially equivalent to Gaussian functions. Using a Gaussian function, the abundance of the *j*th biotic element can be written as:
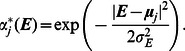
(3)
Equation 3 is the 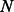-dimensional fundamental niche for the *j*th biotic component. The rate of change of the 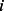
th environmental variable is found with
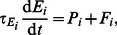
(4)where 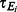 parameterises the timescale of changes in the 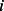
th environmental variable, 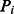 is a perturbation operating on the 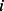
th environmental variable and 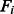 is the sum of the forces the biotic components are exerting on environmental variable 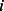. 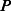 is analogous to insolation in Daisyworld that changed over time due to an increase in the brightness of the star and 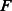 is analogous to the combined albedo of the black and white daisies that led to changes in the planetary albedo and therefore how much temperature changed with changing insolation. In our model the biotic force can be positive or negative according to the random variable 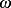, but is always proportional to the abundance of a biotic element. The total biotic force is found by summing the contributions of each component
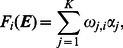
(5)where 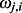 is the effect of the *j*th biotic element on the 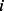
th environmental variable. We are able to describe the model in its entirety with Equations 1, 3, 4 and 5.

## Results

For all results, the force that the *j*th biotic component exerts on the environmental variables, 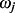, is fixed at an initial random vector with elements chosen uniformly from the range 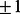. The environmental variable value which maximises the abundance of each biotic component, 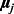 is fixed at an initial random value chosen uniformly from the *essential range*, 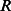, which we set at 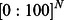. This is the range of environmental conditions that we prescribe are necessary for life. We do not confine or limit possible values for any environmental variable. Therefore in principle any environmental variable may change over time towards 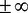. If it is assumed that there are limits to the adaptive capacity of life, then environmental conditions must be bounded in some sense in order for life to survive. This may be understood as the requirement for liquid water to exist on the surface of a planet and so for planetary temperature to be bounded to within (at least) the liquid phase for water. Unlike [14] we do not assume that there is any difference in maximum possible abundance that biotic components are able to achieve within the essential range. For example, a biotic component that has maximum abundance when the environmental variable is five will have the same maximum abundance as a biotic component that has maximum abundance when the environmental variable is near the middle of the essential range at fifty. Having a non-uniform distribution of biotic components across the essential range could affect the results if a system was initialised with environmental variable values a significant distance from any biotic component. Our motivation in using a uniform distribution was not to include any additional assumptions other than life will find a way in that if it is possible for a biotic component to exist within a particular fundamental niche, then it will be present when those conditions are satisfied.

The parameter 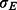 determines the standard deviation of the niche function and so determines the rate of change of abundance as the environmental variable moves away from the optimum value. This is fixed at 5. We will show later that this number is arbitrary and subject to values for the essential range it can be set at any value. The parameters that determine the timescales over which the biotic components and environmental variables change towards to steady state (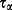 and 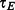 respectively) are set so that the abundances of biotic components do not depart from their environment dependent steady state values (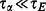). This means that the biotic components are the fastest responding elements within the model. Recently we have shown how assumptions relating to timescales can affect the homeostatic properties of Daisyworld models [36]. Here we assume that biotic components change sufficiently faster than other elements of the model in order to safely ignore the dynamics of how they move towards their steady state values. This greatly facilitates our analysis of the model and so allows us to produce the results pertaining to complexity, stability and how the homeostatic mechanism operates. While relaxing this assumption may lead to changes in the model behaviour under certain circumstances, it does not necessarily mean that the model no longer exhibits homeostasis.

### Rein control homeostasis

**Figure 2:**
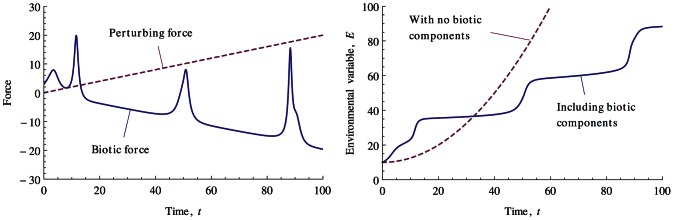
Numerical simulation of a one environmental variable system that contains 100 biotic components, essential range = 100, niche function width = 5. Left: As the perturbing force, 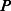 increases over time there is a corresponding opposite sign increase in the force, 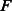, that the biotic components exert on the environmental variable, 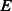. Right: This leads to periods in which the environmental variable remains approximately fixed whereas an abiotic system would see significant increases. Transitions between stable states are characterised by rapid changes in environmental conditions and the biotic force.

Figure 2 shows homeostasis in a numerical simulation featuring a single environmental variable and 100 biotic elements. The environmental variable is initialised at 10. The perturbing force, 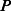, is then linearly increased from zero. In the absence of any feedback by the biotic components on the environmental variable, this would produce an accelerating increase in the environmental variable. The biotic response to this would be a selective sweep through the array of biotic components. Biotic components that had maximum abundance with low environmental variable values would over time be replaced by biotic components that produced maximum abundance with higher and higher environmental variable values. A significant change is observed if biotic components exert randomly initialised effects on the environmental variable. Now the environmental variable remains approximately fixed at a number of values and it is the biotic components' force, 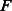, on the environmental variable that changes by taking the same magnitude of the perturbing force, but with the opposite sign. This shares certain features of a control system in that a target value of 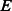 emerges that is regulated in the presence of a perturbing force.

**Figure 3:**
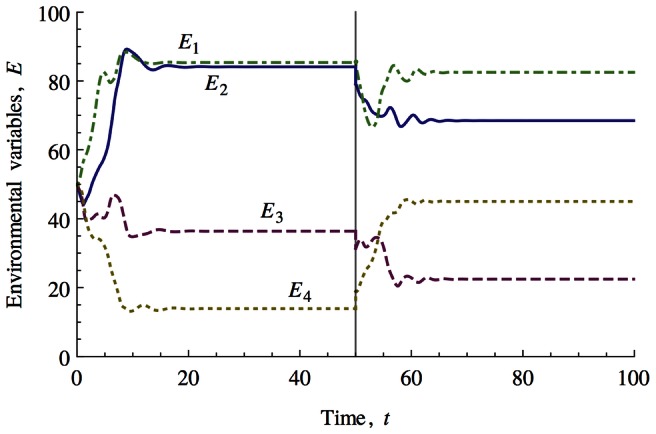
Numerical simulation of a four environmental variable system that contains 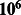 biotic components, essential range = 100, niche function width = 5. All environmental variables are initialised at 50. The system settles into a stable state by time = 20. A ‘shock’ (large, instantaneous perturbation) is applied to the system at time = 50 which moves the system out of the attractor for the homeostatic state and produces large changes in the environmental variables until the system relaxes into another attractor.

Figure 3 shows the numerical simulation results for a typical four environmental variable system that is perturbed by an instantaneous shock and its subsequent recovery to a new stable point. Readers familiar with the operation of Ashby's Homeostat [37] may find this behaviour similar. It is important to note that for all the results and analysis presented here, selection only operates on how adapted the biotic components are to environmental conditions and not the effects the biotic components have on these environmental conditions. However it is the latter that appears to respond to external perturbations with environmental conditions remaining approximately fixed. How are we to understand these results?

The homeostatic mechanism in our model can be regarded as an example of a rein control system. A verbal formulation of rein control was given during the early development of the cybernetics movement by Clynes who proposed that certain physiological processes were modulated by opposing unidirectional affecters [38]. The analogy was made to how a horse is controlled. Two reins are required to steer the horse left and right because reins can only pull not pull and push. In Daisyworld the two reins of black (increasing effect) and white (decreasing effect) daisies regulate temperature [19]. However, as was previously noted for example in [20] the direction of these effects are prescribed and means that a negative feedback loop will be favoured over a range of forcing. For example, if black daisies were to emerge that outcompeted white daisies at lower temperatures but had a decreasing rather than increasing effect on temperature, they would destabilise the system and lead to the extinction of the white daisies and drive temperatures to outside of the essential range. One way to relax this assumption would be to allow the emergence of such destabilising daisies by introducing mutation in the albedo traits of daisies. The consequences of mutation in Daisyworld have been studied previously, for example [39], [40]. Our model is initialised with a random population of biotic components some of which can be regarded as analogues of cold-loving, temperature-decreasing daisies and therefore with the capacity to disrupt homeostatic states. Given that we do not prescribe the traits of the biotic components, one answer to why homeostasis is observed in Figure 2 is that we (the observer) were lucky to see it and that systems that exhibit no homeostatic behaviour are just as probable.

**Figure 4:**
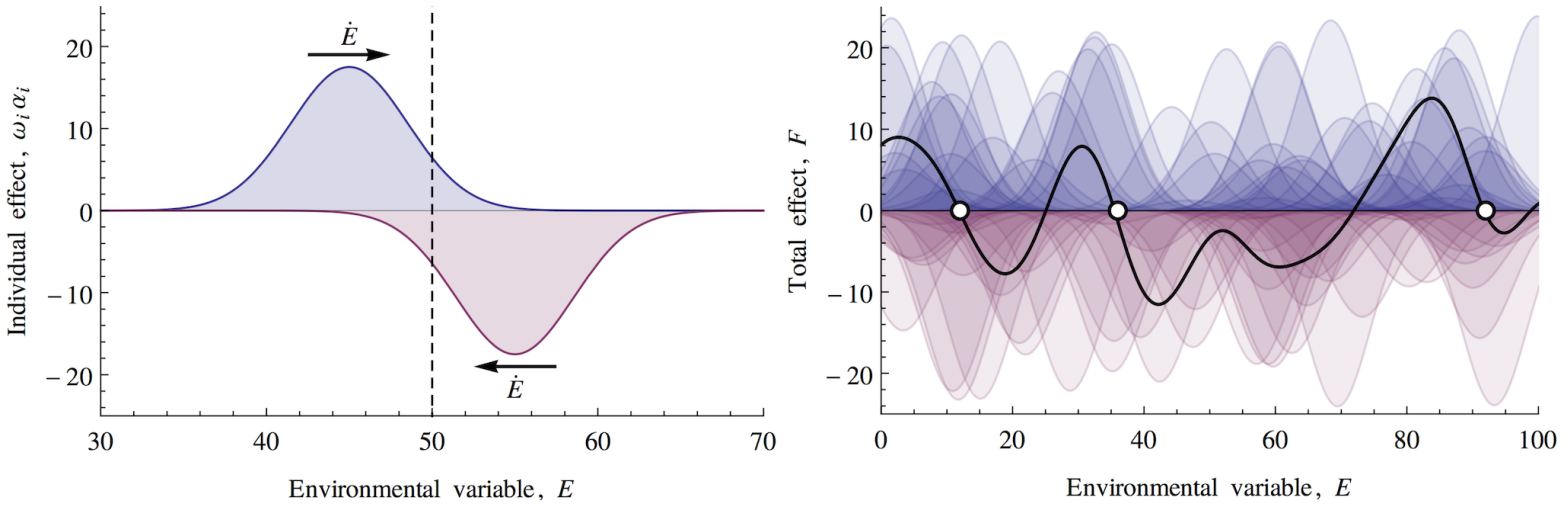
Left: A rein control state is shown. A biotic component that increases the environmental variable, 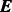, counteracts the effects of a biotic component that decreases 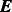. This results in 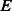 being regulated around values near the vertical dashed line. The probability of such a rein control pair being present in a population of two biotic components is 1/4. Right: As the number of biotic components is increased up to 100 in this example, a total effect 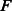 (solid black line) emerges as it is the sum of the individual biotic effects. Homeostatic stable points (denoted with circles) emerge whenever 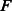 undergoes a zero-crossing from left to right. These correspond to rein control homeostatic states.

Rein control in our model will be established when a biotic component that has an increasing effect on the environmental variable overlaps with a biotic component that has higher abundance at higher values for the environmental variable and has a decreasing effect on the environmental variable. This is represented in Figure 4 Left. In order for the environmental variable to remain bounded within the essential range, an opposing pair of biotic components must emerge. The probability that any two randomly initialised biotic elements will satisfy this condition is 1/4. Figure 4 Right shows that as more biotic components are added, the total effect that they have on the environmental variable will vary and occasionally change sign. The change in sign from positive to negative correspond to rein control states.

### Parameter sensitivity analysis

**Figure 5:**
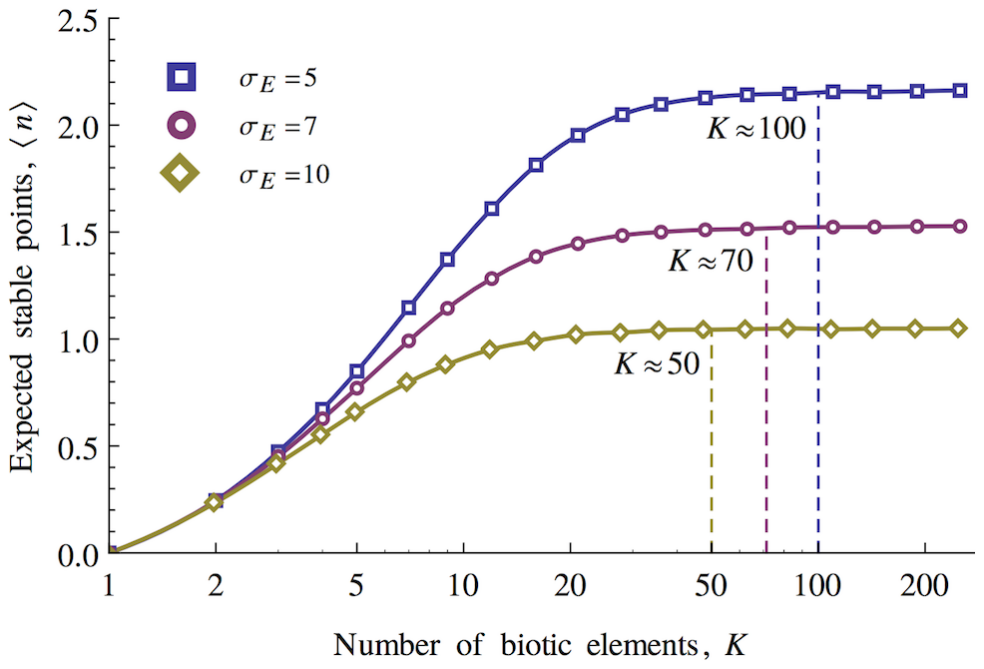
The expected number of stable points for a single environmental variable system with a fixed essential range of 100 increases and then saturates with increasing number of biotic components 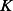 to a value that is dependent on the width of the niche function, 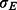.

The force the array of biotic components exert will change as more biotic components are added. At the limit of very large 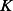 and when Gaussian niche functions are used we find that the expected number of times it changes sign and so the number of stable states in the model is
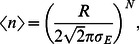
(6)where 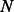 is the number of environmental variables, 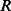 is the essential range and 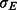 the width of the biotic niche function. See Text S1 for a derivation of these results which takes advantage of the fact that the total effect the biotic components have on the environment, 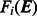, consists of random uncorrelated values associated with each point in 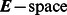. This allows us to use the central limit theorem to show that the expected number of stable states in the model is determined by the ratio of the width of essential range to the width of niche function. This analysis assumes that values of 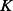 are very large. Numerical simulations allow us to explore the behaviour of the model as 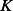 is increased from 1. We find that beyond a threshold value of 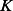, the expected number of times 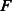 changes sign and so the expected number of stable points remains constant. The threshold value of 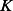 is approximately
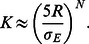
(7)
Figure 5 shows numerical results for a range of 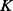 values. Text S2 contains details of how we use a Gaussian process method in order to be able to efficiently compute values for 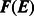 for very large 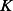.

**Figure 6:**
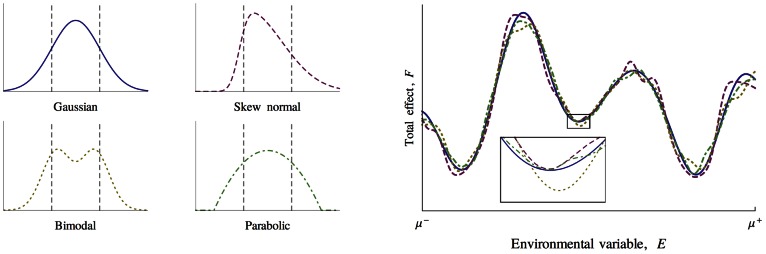
Left: Four niche functions are shown along with their respective characteristic widths (dashed vertical lines). Right: The total biotic force, 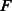, that the different niche functions produce in a population of 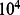 biotic functions is shown. Only the characteristic width and not the particular form of the niche function is important for the establishment of homeostatic states.

Equation (6) and Equation (7) and makes clear the role of the width of niche function, 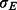, in guiding the model, while the specific function chosen is unimportant. Indeed, as shown in Figure 6, skewed, bimodal and to some extent, fat-tailed functions can be shown to produce similar behaviour. All that matters is that the niche functions are well behaved in the sense of having a characteristic width.

**Figure 7:**
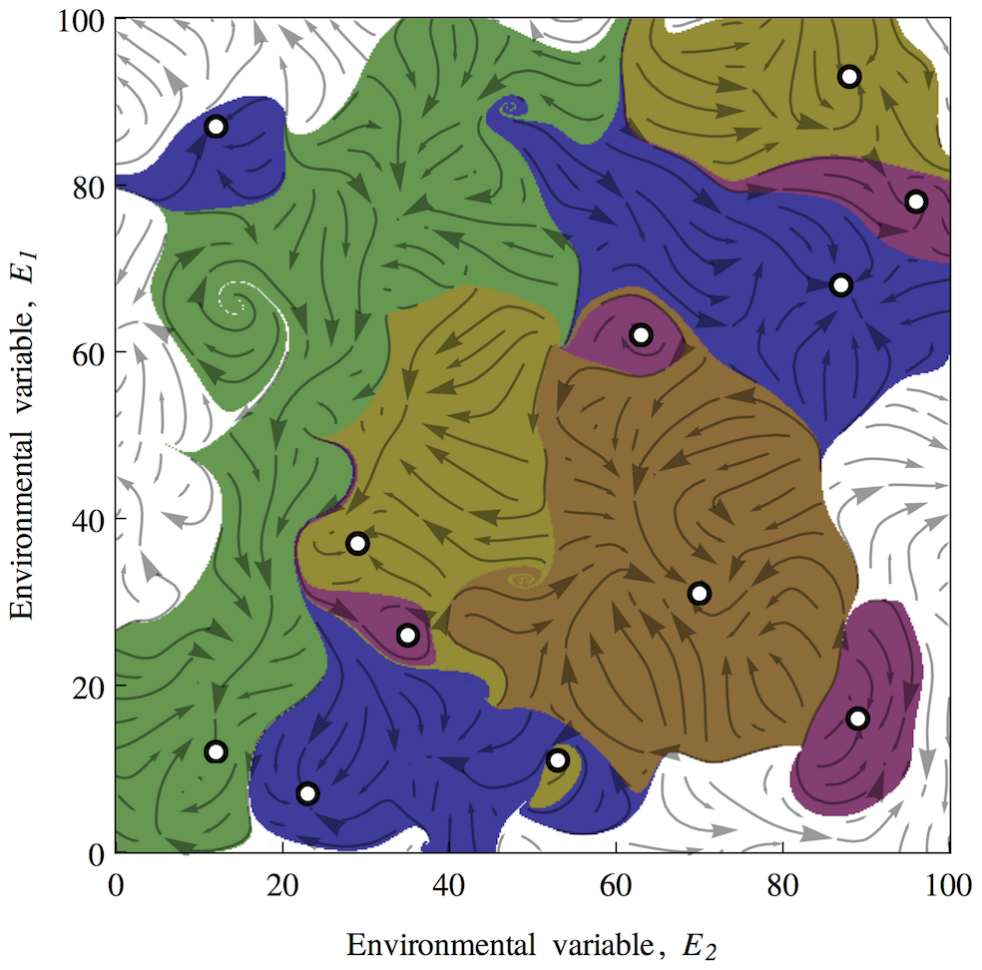
Phase portrait of a two environmental variable system where 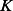 is in the very large limit, essential range = 100, niche function width = 5. Stable points, are indicated by circles. The basins of attraction which lead to these points are indicated by the different coloured enclosing regions, while initial conditions which would leave the essential range of 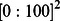 are coloured white. Environmental variables do not necessarily move immediately towards stable points.

**Figure 8:**
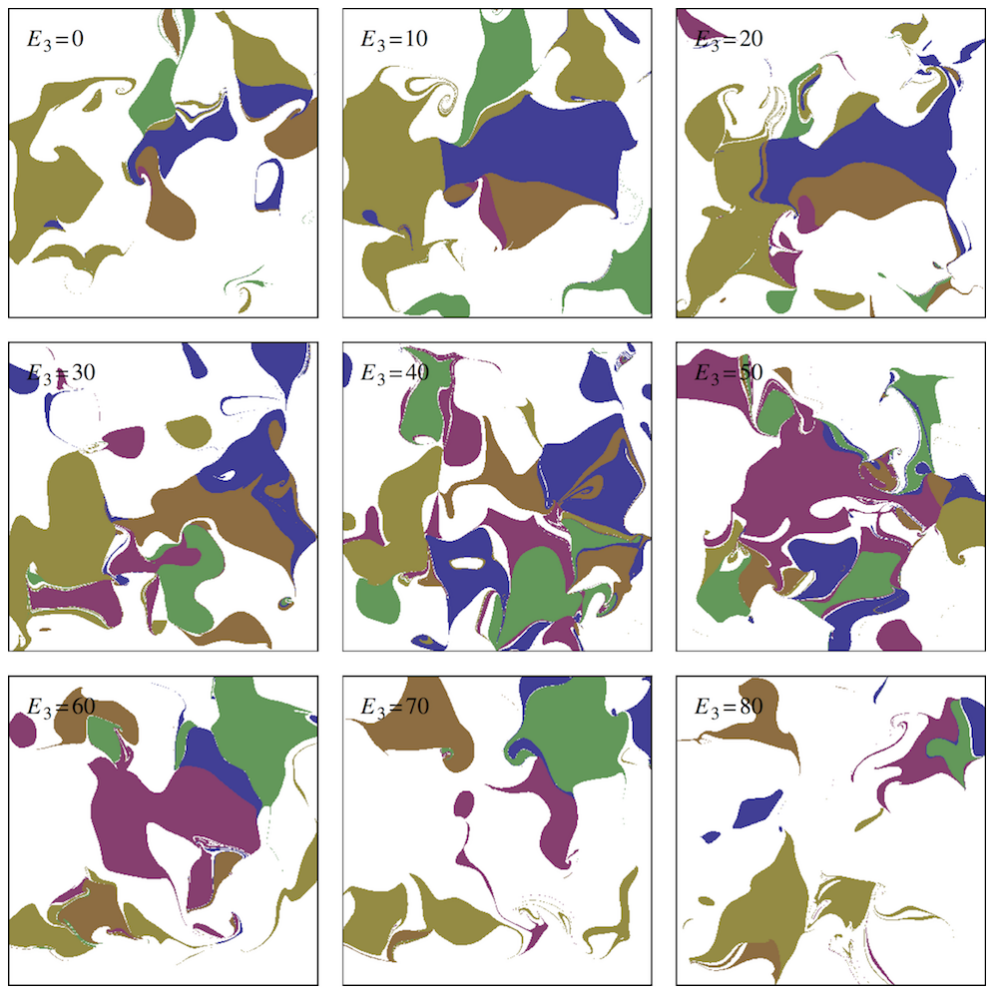
Phase portraits of a three environmental variable system where 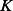 is in the very large limit, essential range = 100, niche function width = 5. Nine slices through the 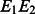-plane are shown with the third environmental variable value increasing from 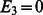 from top left to 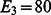 to bottom right.

While Equation 6 tells us there is an exponential increase in the number of stable points as the number of environmental variables increases, numerical results show that as the number of environmental variables is increased, this is accompanied by a decrease in the probability, 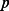, that an environmental variable will remain within the essential range of 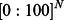 as its moves towards a stable point (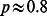 when 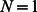 and an approximately linear decrease with increasing 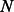 until 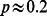 when 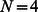). Inspection of phase portraits for higher dimensional systems provides some insights in the qualitive changes in model behaviour with increasing environmental variables. Figure 7 shows the phase portrait for a 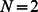 system. The white regions represent starting points whose trajectories leave the essential range of 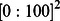. It is important to note that the trajectory of the environmental variables is not necessarily towards the stable points. As well as saddle points between attractors, there can be twists and spirals within a particular attractor. Increasing the number of environmental variables to three increases both the complexity of the attractor landscape and the regions of white space as show in Figure 8.

## Discussion

### Complexity and stability

The relationship between complexity and stability is a well established topic in ecology [41]. An important early paper reported an inverse relationship between diversity and stability in simple model ecosystems [42]. As the number of linear connections between species increased, the probability that the ecosystem would be stable decreased. This finding was in part based on a study that found the stability of large systems underwent a catastrophic collapse at a certain level of connectivity [43]. Our results can be interpreted in the light of these landmark studies. As the number of environmental variables, 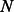, is increased we found an exponential increase in the *number* of stable states to be accompanied by a decreasing *probability* that a randomly initialised system will remain with the essential range as it relaxes towards stable points. Increasing 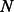 corresponds to an increase in the number of biotic interactions as all such interactions are mediated via environmental variables. A more connected system is less stable in that respect. However, we found that the stability of the model increased with increased biodiversity up to a threshold, beyond which further increases in the numbers of biotic components had no effect on the expected number of stable points. How are we to understand this result in the light of [42]?

First we remind ourselves that there are no inter-biotic competitive or trophic interactions. However, given that all biotic components affect environmental conditions that in turn affect all biotic components, there is in that sense a system with a high degree of connectivity. Given the explanation of a rein control system in Section “Rein control homeostasis”, one may assume that during periods of homeostasis, there is a significant reduction in the degree of connectivity as most biotic components would have negligible abundance and so minimal impacts on the stability of the system. Such homeostatic states emerged in [20], [44] due to the presence of strong inter-species competition. This is not the case in our model as is demonstrated in Figure 3 Right. For any stable point, there will be a ‘cloud’ of biotic components as there is no competitive exclusion operating. This picture becomes further complicated in higher dimensions as there will be further clouds of biotic components at other points along any particular axis.

**Figure 9:**
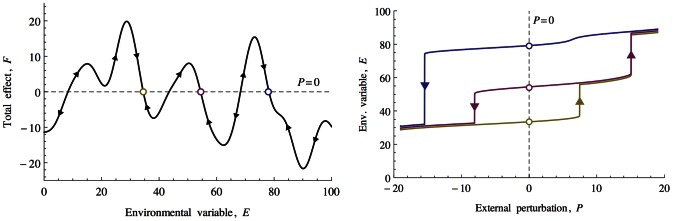
Left: The model has homeostatic points (denoted with circles), where the total biotic effects changes sign from positive to negative. Right: Hysteresis is caused by the existence of multiple homeostatic points for a given external perturbation. The yellow, red and blue stable points correspond to the coloured circles in the left figure. Recovery back to the red state after a transition to the blue state as a result of increasing 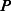 is only possible via a large decrease in 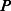 and a transition via the yellow state.

A crucial difference between our model and studies such as [42] is that we do not assume that interactions are linear or monotonic. Our initial assumption is that the fundamental niche of any biotic component is finite. For any environmental axis there will be conditions that are too low or too high. The next parsimonious assumption would be that the abundance of any biotic component would decrease towards these extremes from a central region. That is, there is an inflection in abundance over the fundamental niche. We showed how the particular form of the biotic's response function is irrelevant to the overall establishment of homeostasis. All that matters is that it is ‘well behaved’ in the sense of being bounded and therefore having a characteristic width. This explains one conclusion of [45] which found that the width of response functions can tend to infinitesimally narrow with no change in observed homeostasis. Biotic components with niche functions that feature one or more inflections allow a system to transition from positive feedback loops to negative feedback loops and vice versa. Consider Figure 9 Left. If an external perturbing force is sufficiently great to drive the environmental variable away from a stable point over an inflection in the total biotic force, then the previous negative feedback loop immediately transitions to a positive feedback loop that will quickly drive the environmental variable to a new stable point. This explains the sharp transitions between homeostatic states and the hysteresis loops shown in Figure 9 Right. The bounded niche functions are at the root of how increasing the complexity of the model by increasing the number of biotic components does not, beyond a threshold of biodiversity, decrease the probability of it establishing a stable point.

### Limitations

The original Daisyworld was conceived as a proof of concept for a homeostatic biosphere. The model presented here revisits the original Daisyworld and a number of its variants and identifies those conditions that are required for homeostasis to emerge. These conditions are arguably the most parsimonious in that all that is required is that life is affected by environmental conditions which in turn are affected by life. Of course, this leaves a great deal of detail either opaque or absent. For example, recently we have argued that it is important to consider timescales in models of environmental homeostasis [36]. For all the results presented here, the parameters that determined the timescale over which the biotic components and environmental variables evolved towards a steady state, were fixed such that the biotic components changed over much faster timescales than environmental variables. For certain aspects of the Earth system this may be a reasonable assumption. For example the heat content of the Earth's oceans changes over timescales of thousands of years during which many generations of species may have lived. However, not all biotic components respond significantly faster than all environmental variables on Earth. It has been found that relaxing a number of assumptions relating to timescales in the original single environmental variable Daisyworld introduces more complex behaviour, and under a range of conditions could significantly reduce the homeostatic properties of the model [36]. It also significantly complicates analysis, and given that an important motivation of this study was to identify general features of multi-environmental variable systems, explains why timescales were not analysed here. However, it is an important issue to explore further as our study assumes that the system operates at maximum fidelity with respect to how much information is transmitted between the components. In order for homeostasis to be observed, the biotic components must ‘know’ what the state of the environment is and act quickly in the appropriate fashion. Any attenuation in a signal from environment to the biota and back again introduces potentially important lags.

Another significant limitation of the model is that the traits of the biotic components once initialised are fixed. Consequently the model does not feature any adaptive, acclimation or evolutionary processes. An early criticism of the original Daisyworld model was that such a form of Gaian homeostasis would not be robust to ‘cheats’ that would destabilise the system [5]. The biotic components were not part of an evolutionary stable strategy and invasion of the strategy by mutants would destabilise and ultimately collapse any form of environmental homeostasis. Subsequently, this subject has been discussed at some length (see [6], [18] for reviews) and has led to the conception of Gaia as a self-organising system comprised of organismic by-products [46]. Our model may be understood as an ecotype model [47] in which microbial species, which are the key waste recyclers in the biosphere, are components in an emergent system of negative feedback recycling loops. If environmental altering effects are incidental by-products (such as the excretion of metabolic waste products) then it is not necessary for environmental altering traits to confer any advantage for the organisms that possess them. What will often arise in our model system is a situation in which biotic components exert forces on environmental variables that would, in the absence of other biotic forces, move the environmental variable further *away* from the value that produces maximum abundance. There need be no selective advantage to the biotic components in order for environmental homeostasis to emerge and be maintained. However, it is still to be expected that relaxing the assumption that all biotic component traits remain fixed would introduce potentially important new behaviour. Here we note that the single environmental variable version of this model is conceptually very similar to the model detailed in [20] that allowed progressive mutations in the effects biotic components had on environmental variables and how the abundance of biotic components was affected by environmental variables. It was shown that inclusion of these assumptions did not significantly alter the homeostatic behaviour of the model.

### Conclusions

We have shown that homeostasis can emerge in a system that featured a diverse array of biological components and multiple environmental variables. Assuming that all ecological niches can be potentially occupied results in at least one homeostatic state being realised in the system. Homeostasis in our system is a consequence of the sum of forces the individual biotic components have on the environmental variables. These forces increase, decrease and can change sign. When the biotic force changes from positive to negative a homeostatic state is produced that is robust to a range of perturbations. Gaussian processes analysis showed that increasing the number of biotic components does not, above a certain threshold, decrease the probability that the system will be stable and homeostatic. A more diverse system is not less stable than a less diverse system. This property of the model can be understood in the light of Ashby's law of requisite variety that stated a controller must be able to produce at least as many different configurations as demanded by external perturbing forces [37]. In our model there must be sufficient amounts of biodiversity to produce a total biotic force that will undergo at least one change in sign.

Increasing the number of environmental variables led to an exponential increase in the number of stable points, while leading to an approximately linear decrease in the probability that environmental variables in a randomly initialised system will remain within a prescribed essential range of values. In that respect an increase in model complexity led to a reduction in the probability that the system would be stable. Given that the current day Earth was not instantaneously formed in its current state but has evolved from simpler configurations, it is interesting to speculate how robust this relationship between environmental complexity and stability is in our model if model complexity was incremented via adding additional environmental variables to stable systems. Such an approach would be similar to [48] that found sequential selection may provide an account for the emergence and persistence of homeostatic complex systems.

## Supporting Information

Figure S1 The solution to Equation S1.9 may be found by determining the fraction of the unit circle which satisfies the spherically symmetric constraint indicated.
(TIFF)

Figure S2The vector ***Z*** contains function values corresponding to the 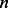 grid points, 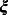. These may then be simply interpolated to approximate the function.(TIFF)

Text S1 Derivation of the number of fixed points with increasing numbers of biotic components.
(PDF)

Text S2 Numerical methods for calculation of the total biotic force.
(PDF)
