# Supplementary figures and images for: The Emergence of Environmental Homeostasis in Complex Ecosystems
Source: PLoS Comput Biol. 2013 May 16;9(5):e1003050. doi: 10.1371/journal.pcbi.1003050 (PMC3656095; doi:10.1371/journal.pcbi.1003050)

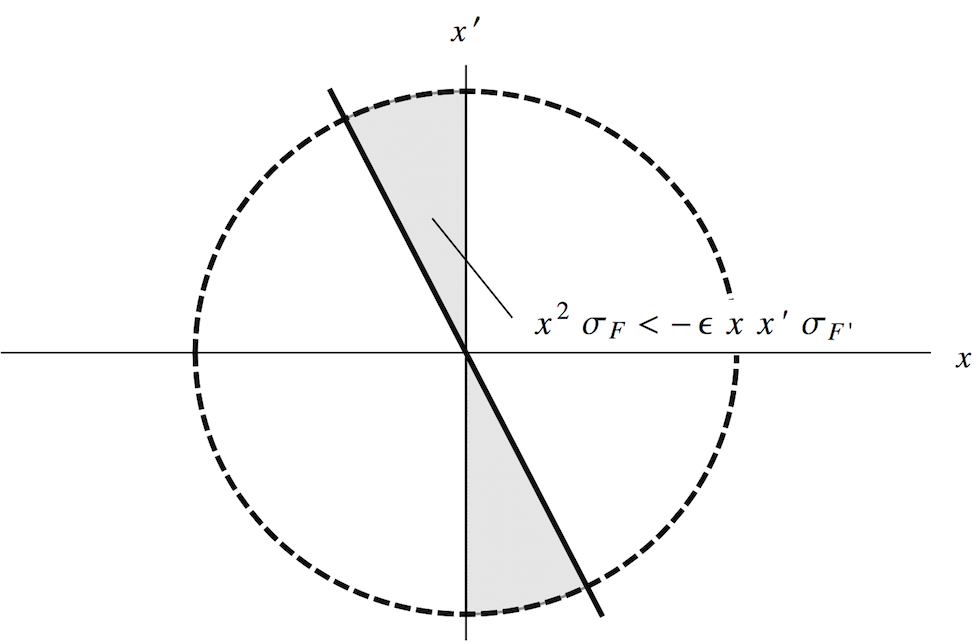

Supplement: Figure S1 — The solution to Equation S1.9 may be found by determining the fraction of the unit circle which satisfies the spherically symmetric constraint indicated. (TIFF) [file pcbi.1003050.s001.tiff]

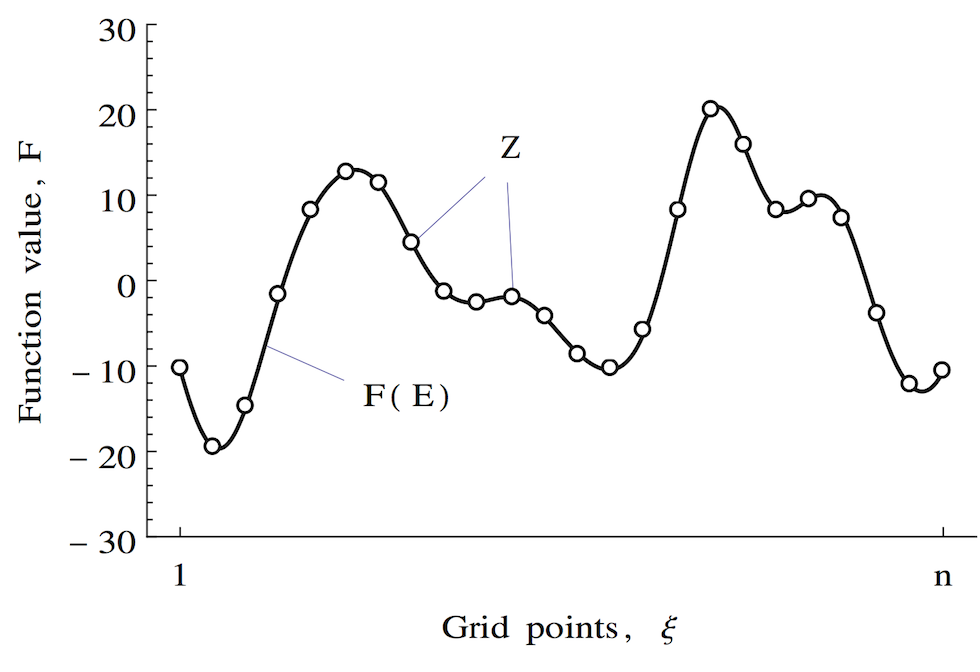

Supplement: Figure S2 — The vector Z contains function values corresponding to the grid points, . These may then be simply interpolated to approximate the function. (TIFF) [file pcbi.1003050.s002.tiff]
