# Supplementary material for: The Emergence of Environmental Homeostasis in Complex Ecosystems
Source: PLoS Comput Biol. 2013 May 16;9(5):e1003050. doi: 10.1371/journal.pcbi.1003050 (PMC3656095; doi:10.1371/journal.pcbi.1003050)
Supplement: Text S1 — Derivation of the number of fixed points with increasing numbers of biotic components. (PDF) [file pcbi.1003050.s003.pdf]

The emergence of environmental homeostasis in complex ecosystems: Supporting Information 1

James G. Dyke*, Iain S. Weaver
School of Electronics and Computer Science, University of Southampton, University Road, Southampton, SO17 1BJ
* email: jd4@ecs.soton.ac.uk

We begin analysis by deriving the covariance function of the total biotic force $\boldsymbol{F}$ in terms of the biotic response functions $\alpha(\boldsymbol{E})$. The following analysis is much simpler under the approximation that the function $\boldsymbol{F}(\boldsymbol{E})$ is *stationary*, which assumes the limit $R \gg \sigma_E$ where the statistics of $\boldsymbol{F}(\boldsymbol{E})$ are uniform within the essential range. In reality, close to the edges of the essential range the amplitude of $\boldsymbol{F}(\boldsymbol{E})$ is reduced due to lower total biotic abundance. A consequence of this approximation is that the covariance of $F_i(\boldsymbol{E})$, $k_i(\boldsymbol{E}, \boldsymbol{E}')$, is also stationary, and depends only on the distance $|\boldsymbol{E} - \boldsymbol{E}'| = \Delta\boldsymbol{E}$

$$k_i(\Delta\boldsymbol{E}) = \left\langle \sum_{n,m=1}^{K} \omega_{i,n}\omega_{i,m}\alpha_n(\boldsymbol{E})\alpha_m(\boldsymbol{E}+\Delta\boldsymbol{E}) \right\rangle. \quad (1)$$

At this point, we can exploit the absence of correlations first between individual biotic components, and then between the weights ω, and the biotic abundance. The first observation leads us to conclude the off-diagonal terms, where $i \neq j$, do not contribute to the covariance. The second enables us to separate the expectation values of ω and α, giving

$$k_i(\Delta\boldsymbol{E}) = K\sigma_\omega^2 \left\langle \alpha(\boldsymbol{E})\alpha(\boldsymbol{E}+\Delta\boldsymbol{E}) \right\rangle. \quad (2)$$

where σ_ω^2 is the variance of the random variable ω. The right side of this equation can be identified simply as the covariance of the individual biotic activity functions. This result illustrates that the covariance of the summed functions share the functional form of the individual functions of which it is comprised. The characteristic length is *independent* of the value of K and therefore the propensity for $\boldsymbol{F}$ to form homeostatic stable points is unaffected by the the biotic complexity of the model.

Next, we aim to derive an expression for the expected number of homeostatic fixed points within the essential range. We begin by stating that a fixed point in $F_i(\boldsymbol{E})$ occurs in a small interval of $\boldsymbol{E}$ if its sign changes across the interval - a sign change from negative to positive would be unstable, while positive to negative results in a homeostatic stable point. Labelling the interval $\boldsymbol{\epsilon}$, this condition can be expressed

$$F_i(\boldsymbol{E})F_i(\boldsymbol{E}+\boldsymbol{\epsilon}) < 0 \quad (3)$$

and the expected number of such points in the unit interval, n_0, is found from a product of indicator functions of the form of Equation (3)

$$n_0 = \left\langle \prod_{i=1}^{N} \frac{1}{\epsilon} \left[F_i(\boldsymbol{E})F_i(\boldsymbol{E}+\boldsymbol{\epsilon}) < 0\right] \right\rangle. \quad (4)$$

We have used square brackets $[\ldots]$ to denote the indicator function

$$[F_i(\boldsymbol{E})F_i(\boldsymbol{E}+\boldsymbol{\epsilon}) < 0] = \begin{cases} 0 & F_i(\boldsymbol{E}) \text{ and } F_i(\boldsymbol{E}+\boldsymbol{\epsilon}) \text{ have same sign} \\ 1 & F_i(\boldsymbol{E}) \text{ and } F_i(\boldsymbol{E}+\boldsymbol{\epsilon}) \text{ opposite sign} \end{cases} \quad (5)$$

The expectation of an indicator function may be interpreted as the *probability* of its contents being true, and the product of several therefore gives the probability of many conditions being met simultaneously. Each term in the product may be treated independently due to the independence between the biotic

effects on the different environmental variables F_i and F_j. The problem is therefore reduced to finding the value of the series of N expectation values. Expanding for small ϵ gives

$$p = \left\langle \left[F_i(\boldsymbol{E})^2 < -\epsilon F_i(\boldsymbol{E}) F_i'(\boldsymbol{E})\right] \right\rangle \tag{6}$$

where $F_i'(\boldsymbol{E})$ is used to indicate the derivative of $F_i(\boldsymbol{E})$ in the $\hat{\boldsymbol{\epsilon}}$ direction

$$F_i'(\boldsymbol{E}) \equiv \nabla_{\hat{\boldsymbol{\epsilon}}} F_i(\boldsymbol{E}).$$

To find the expectation value of this indicator function, we need to know how $F_i(\boldsymbol{E})$ and $F_i'(\boldsymbol{E})$ are distributed. Rather than suffer any loss of generality, we make three important observations;

(i) at any point in $\boldsymbol{E}$ within the essential range, $F_i(\boldsymbol{E})$ is a sum of *independent* contributions from the biotic elements. Therefore, by the central limit theorem, each point follows a Gaussian distribution.

(ii) this distribution has a mean of zero as previously stated. There is no tendency for positive or negative feedback between the biota and environment.

(iii) $F_i(\boldsymbol{E})$ and $F_i'(\boldsymbol{E})$ to be uncorrelated as a consequence of our independent parameters $\boldsymbol{\mu}$ and $\boldsymbol{\omega}$.

The problem is now dramatically reduced, we need only find the variance of the Gaussian random variables $F_i(\boldsymbol{E})$ and $F_i'(\boldsymbol{E})$, labelled σ_F^2 and $\sigma_{F'}^2$ respectively. Having already determined the covariance of $F_i(\boldsymbol{E})$ in Equation S1:1, the variance therefore may be written as

$$\sigma_F^2 = k_i(\mathbf{0}). \tag{7}$$

We can write a similar expression for $F_i(\boldsymbol{E})$, and remove the directional derivative from the expectation value to give

$$\begin{aligned}\sigma_{F'}^2 &= \left\langle F'(\boldsymbol{E}) F'(\boldsymbol{E}') \right\rangle \big|_{\boldsymbol{E}=\boldsymbol{E}'} \\ &= \nabla_{\boldsymbol{E},\hat{\boldsymbol{\epsilon}}} \nabla_{\boldsymbol{E}',\hat{\boldsymbol{\epsilon}}} k_i(\boldsymbol{E} - \boldsymbol{E}') \big|_{\boldsymbol{E}=\boldsymbol{E}'} \\ &= -k_i''(\mathbf{0}).\end{aligned} \tag{8}$$

Next, we substitute Equations (7) and (8) into Equation (6)

$$p = \iint \left[F_i(\boldsymbol{E})^2 < -\epsilon F_i(\boldsymbol{E}) F_i'(\boldsymbol{E})\right] P(F_i) P(F_i') \mathrm{d}F_i \mathrm{d}F_i' \tag{9}$$

where $P(F_i)$ and $P(F_i')$ are the Gaussian distributions

$$P(F_i) = \frac{1}{\sqrt{2\pi}\sigma_F} \exp\left(-\frac{F_i^2}{2\sigma_F^2}\right), \tag{10}$$

$$P(F_i') = \frac{1}{\sqrt{2\pi}\sigma_{F'}} \exp\left(-\frac{F_i'^2}{2\sigma_{F'}^2}\right). \tag{11}$$

After a change of variable, $\frac{F_i}{\sigma_F} \to x$ and $\frac{F_i'}{\sigma_{F'}} \to x'$, we can exploit spherical symmetry in x and x', illustrated in Figure S1.

$$p = \iint \left[x^2 \sigma_F < -\epsilon x\, x' \sigma_{F'}\right] \frac{e^{-\frac{x^2}{2}}}{\sqrt{2\pi}} \frac{e^{-\frac{x'^2}{2}}}{\sqrt{2\pi}} \mathrm{d}x \mathrm{d}x' \tag{12}$$

We need only find the fraction of the $x\,x'$ plane for which $x^2\sigma_F < -\epsilon x\,x'\sigma_{F'}$. We therefore find the expectation of the indicator function to be

$$p = \frac{1}{\pi}\,\mathrm{atan}\left(\epsilon\frac{\sigma_{F'}}{\sigma_F}\right) \tag{13}$$

which can be expanded to first order for small ϵ, and substituted into Equation (4) to give

$$n_0 = \left(\frac{p}{\epsilon}\right)^N = \left(\frac{1}{\pi}\sqrt{-\frac{k''(\mathbf{0})}{k(\mathbf{0})}}\right)^N, \tag{14}$$

which is consistent with [1, Theorem 4.1.1] for the case of a one-dimensional mode. Counter to intuition, as the number of environmental variables increases the number of stable fixed points within the essential range of the model may *increase* exponentially, rather than vanishing to zero providing there is a sufficiently wide essential range.

References

1. Alder A, Strassen V (1981) On the algorithmic complexity of associative algebras. Theoretical Computer Science 15: 201–211.
