# Supplementary material for: The Emergence of Environmental Homeostasis in Complex Ecosystems
Source: PLoS Comput Biol. 2013 May 16;9(5):e1003050. doi: 10.1371/journal.pcbi.1003050 (PMC3656095; doi:10.1371/journal.pcbi.1003050)
Supplement: Text S2 — Numerical methods for calculation of the total biotic force. (PDF) [file pcbi.1003050.s004.pdf]

The emergence of environmental homeostasis in complex ecosystems: Supporting Information 2

James G. Dyke*, Iain S. Weaver
School of Electronics and Computer Science, University of Southampton, University Road, Southampton, SO17 1BJ
* email: jd4@ecs.soton.ac.uk

Computation of the total biotic force becomes computationally expensive for large K, typically necessary for interesting behaviour in a higher dimensional environment. To overcome this, the limit $K \to \infty$ is taken, where the function $\boldsymbol{F}(\boldsymbol{E})$ may be represented by its covariance.

Firstly, we devise a spatial grid of n points in the space of environmental variables where we aim to sample the function $\boldsymbol{F}(\boldsymbol{E})$, and will interpolate between. The exact value of n should be chosen carefully as a trade-off between the quality of representation of $\boldsymbol{F}(\boldsymbol{E})$, and the computational cost (and memory requirement) of the matrix decomposition, which scales as $\mathcal{O}(n^2)$. The spatial grid $\boldsymbol{\xi}$ and corresponding function samples, $\boldsymbol{Z}$, are illustrated by Figure S2 and can be written as

$$\boldsymbol{\xi} = \begin{pmatrix} \boldsymbol{E}_1 \\ \boldsymbol{E}_2 \\ \vdots \\ \boldsymbol{E}_n \end{pmatrix} \qquad \boldsymbol{Z} = \begin{pmatrix} \boldsymbol{F}(\boldsymbol{E}_1) \\ \boldsymbol{F}(\boldsymbol{E}_2) \\ \vdots \\ \boldsymbol{F}(\boldsymbol{E}_n) \end{pmatrix} \tag{1}$$

Next, the covariance of $\boldsymbol{F}(\boldsymbol{E})$ between each element of $\boldsymbol{\xi}$ is computed in the so-called *covariance matrix*.

$$\boldsymbol{C} = \begin{pmatrix} k(\boldsymbol{E}_1, \boldsymbol{E}_1) & k(\boldsymbol{E}_2, \boldsymbol{E}_1) & \cdots & k(\boldsymbol{E}_n, \boldsymbol{E}_1) \\ k(\boldsymbol{E}_1, \boldsymbol{E}_2) & k(\boldsymbol{E}_2, \boldsymbol{E}_2) & \cdots & k(\boldsymbol{E}_n, \boldsymbol{E}_2) \\ \vdots & \vdots & \ddots & \vdots \\ k(\boldsymbol{E}_1, \boldsymbol{E}_n) & k(\boldsymbol{E}_2, \boldsymbol{E}_n) & \cdots & k(\boldsymbol{E}_n, \boldsymbol{E}_n) \end{pmatrix} \tag{2}$$

The bulk of the computation is in the Cholesky decomposition of $\boldsymbol{C}$, that is finding the matrix $\boldsymbol{A}$ where

$$\boldsymbol{C} = \boldsymbol{A}^{\mathrm{T}} \boldsymbol{A}. \tag{3}$$

Importantly, while very costly to compute, calculation of $\boldsymbol{A}$ is deterministic and need only be carried out once in order to produce a large number of different functions $\boldsymbol{F}(\boldsymbol{E})$ for numerical validation.

Finally, a matrix $\boldsymbol{W}$ of independent random values is generated corresponding to each of the n grid points and the N environmental variables,

$$\begin{matrix} & F_1 & F_2 & \ldots & F_N & \\ \boldsymbol{W} = & \left(\begin{matrix} w_{1,1} \\ w_{1,2} \\ \vdots \\ w_{1,n} \end{matrix} \right. & \begin{matrix} w_{2,1} \\ w_{2,2} \\ \vdots \\ w_{1,n} \end{matrix} & \begin{matrix} \ldots \\ \ldots \\ \ddots \\ \ldots \end{matrix} & \left. \begin{matrix} w_{N,1} \\ w_{N,2} \\ \vdots \\ w_{N,n} \end{matrix} \right) & \begin{matrix} \boldsymbol{\xi}_1 \\ \boldsymbol{\xi}_2 \\ \vdots \\ \boldsymbol{\xi}_n \end{matrix}, \end{matrix} \tag{4}$$

and the sampling points $\boldsymbol{Z}$ are computed by the vector product of $\boldsymbol{A}$ with $\boldsymbol{W}$. The function $\boldsymbol{F}(\boldsymbol{E})$ is found by basic interpolation between grid points, illustrated by Figure S2.

$$\boldsymbol{Z} = \boldsymbol{A} \cdot \boldsymbol{W} \tag{5}$$
